# Bone Marrow-Sparing IMRT in Anal Cancer Patients Undergoing Concurrent Chemo-Radiation: Results of the First Phase of a Prospective Phase II Trial

**DOI:** 10.3390/cancers12113306

**Published:** 2020-11-09

**Authors:** Francesca Arcadipane, Patrick Silvetti, Francesco Olivero, Alessio Gastino, Viola De Luca, Massimiliano Mistrangelo, Paola Cassoni, Patrizia Racca, Elena Gallio, Adriana Lesca, Christian Fiandra, Umberto Ricardi, Pierfrancesco Franco

**Affiliations:** 1 Department of Oncology, Radiation Oncology, Azienda Ospedaliero-Universitaria Citta’ della Salute e della Scienza, 10126 Turin, Italy; francesca.arcadipane@gmail.com; 2 Department of Oncology, Radiation Oncology, University of Turin, 10126 Turin, Italy; patrick.silvetti@edu.unito.it (P.S.); francesco.olivero668@edu.unito.it (F.O.); alessio.gastino@gmail.com (A.G.); viola.deluca89@gmail.com (V.D.L.); christian.fiandra@unito.it (C.F.); umberto.ricardi@unito.it (U.R.); 3 Department of Surgical Sciences, Abdominal Surgery, University of Turin, 10126 Turin, Italy; mistrangelo@katamail.com; 4 Department of Medical Sciences, Pathology Unit, University of Turin, 10126 Turin, Italy; paola.cassoni@unito.it; 5 Department of Oncology, Oncological Centre for Gastrointestinal Neoplasm, AOU Città della Salute e della Scienza, 10126 Turin, Italy; pracca@cittadellasalute.to.it; 6 Medical Physics Unit, S.C. Fisica Sanitaria, A.O.U. Città della Salute e della Scienza, 10126 Turin, Italy; gallio.elena@gmail.com; 7 Division of Nuclear Medicine, AOU Città della Salute e della Scienza, 10126 Turin, Italy; alesca@cittadellasalute.to.it

**Keywords:** anal cancer, bone marrow-sparing IMRT, hematologic toxicity, radiotherapy

## Abstract

**Simple Summary:**

Hematological toxicity may be a consistent issue in anal cancer patients undergoing concurrent chemo-radiation, with a potentially detrimental effect on clinical outcomes and patient compliance to treatment. Chemotherapy is the most important trigger, since it induces myelosuppression, but radiation dose delivered to the hematopoietically active bone marrow (BM) also plays an important role. Active bone marrow can be identified using functional imaging with 18-Fluoro-2-deoxy-glucose positron emission tomography (^18^FDG-PET) and selectively spared during radiation delivery via intensity-modulated radiotherapy (IMRT). We investigated, within a prospective phase II trial, the potential effectiveness of targeted avoidance of active BM comprised within pelvic bones in reducing the acute hematologic toxicity profile of anal cancer patients undergoing concomitant chemo-radiation for squamous cell carcinoma of the anus. The results of the first step of the study fulfilled the criteria to define BM-sparing IMRT as “promising” and to continue with the second step of the phase II trial.

**Abstract:**

Purpose: to investigate the role of selective avoidance of hematopoietically active BM within the pelvis, as defined with ^18^FDG-PET, employing a targeted IMRT approach, to reduce acute hematologic toxicity (HT) profile in anal cancer patients undergoing concurrent chemo-radiation. Methods: a one-armed two-stage Simon’s design was selected to test the hypothesis that BM-sparing approach would improve by 20% the rate of G0–G2 (vs. G3–G4) HT, from 42% of RTOG 0529 historical data to 62% (α = 0.05 and the β = 0.20). At the first stage, among 21 enrolled patients, at least 9 should report G0–G2 acute HT to further proceed with the trial. We employed ^18^FDG-PET to identify active BM within the pelvis. Acute HT was assessed via weekly blood counts and scored as per the Common Toxicity Criteria for Adverse Effects version 4.0. Results: from December 2017 to October 2019, 21 patients were enrolled. Maximum observed acute HT comprised 9% rate of ≥G3 leukopenia and 5% rate of ≥G3 neutropenia and anemia. Overall, only 4 out of 21 treated patients (19%) experienced ≥G3 acute HT. Conversely, 17 patients (81%) experienced G0–G2 events, way above the threshold set by the trial design. Conclusion: ^18^FDG-PET-guided BM-sparing IMRT was able to reduce acute HT in anal cancer patients treated with concomitant chemo-radiation. These results prompted us to conclude the second part of this prospective phase II trial.

## 1. Introduction

Combined modality treatment including radiotherapy (RT) and chemotherapy (CHT) administered concurrently is the standard curative approach for patients affected with squamous cell carcinoma of the anal canal [1]. This approach provides high rates of tumor control and patient’s survival, together with the possibility to preserve the anal sphincter [2]. Nevertheless, the toxicity profile of concurrent RT-CHT is relevant and may lead to impaired patients compliance to therapy with subsequent unscheduled treatment breaks and increased overall treatment time which may affect clinical outcomes [3]. This is particularly evident when RT is delivered with conventional techniques, as shown by the rate of grade 3–4 toxicity events seen in the 5-fluorouracil/mytomicin C arm of the RTOG 9811 trial, where major skin toxicities were as high as 48%, while hematologic toxicity up to 61% [3]. Intensity-modulated radiotherapy (IMRT), a technique able to improve the conformity of radiation dose distribution compared to 3-dimensional conformal radiotherapy, was shown to reduce the rates of ≥G3 acute gastro-intestinal and skin toxicity and the likelihood to experience ≥ G2 hematologic events, as seen in the RTOG 0529 study [4]. However, even with the use of highly conformal techniques, toxicity remains clinically meaningful and its reduction deserves targeted strategies [5]. In this subset of patients, hematologic toxicity (HT) can be critical, increasing the likelihood to experience bleeding, infections or asthenia and potentially hampering the overall treatment intensity [6]. CHT is the strongest trigger for HT, given its direct myelosuppressive action, but also RT, given the exquisite radiosensitivity of circulating blood cells and precursors within bone marrow (BM), plays a role [6]. It has been previously demonstrated that the dose received by the pelvic bones, either as outlined employing the outer contour on computed tomography or by the hematopoietically active BM, as defined with the use of ^18^fluorodeoxyglucose (FDG)-labeled positron emission tomography (^18^FDG-PET) is significantly correlated with the probability of occurrence and the severity of HT in anal cancer patients undergoing concurrent RT-CHT [7,8,9,10,11]. Active BM comprised within the pelvic region may be used as an organ at risk to be taken into account during the optimization process of RT planning in order to minimize the unintended dose received and consequently spare hematopoietic precursors.

To test the hypothesis that the selective sparing of hematopoietically active BM may decrease the acute HT profile in anal cancer patients undergoing concurrent RT-CHT, we designed and ran a prospective phase II trial. We herein report on the results of the first phase of the study.

## 2. Material and Methods

### 2.1. Eligibility Criteria

All patients enrolled had a histologically proven diagnosis of squamous cell anal carcinoma involving either the anal canal or margin, obtained with a punch biopsy during fiber-optic endoscopic examination. Disease stage, based on pelvic magnetic resonance, chest-computed tomography, and whole-body ^18^FDG-PET, was defined following the 7th Edition of the American Joint Committee on Cancer staging manual [12]. Patients included were staged as T1–T4, N0–N3, and treated within the Radiation Oncology Department of the University of Turin. Other inclusion criteria comprised an Eastern Cooperative Oncology Group (ECOG) performance status of 0–1, age 18–80, suitable hematological parameters (neutrophils ≥ 1.5 × 10^9^/L and platelets ≥ 100 × 10^9^/L), adequate renal and liver functions. Exclusion criteria included systemic spread at presentation, prior pelvic radiotherapy, medical contraindications to combination therapy, malabsorption syndrome, peripheral neuropathy, psychiatric disease hampering compliance to therapy, pregnancy, and breast-feeding. Patients with a T1 epidermoid tumor of the anal margin were excluded, being this setting considered to be a different clinical entity. Written informed consent was obtained for all patients.

The study was conducted in accordance with the Declaration of Helsinki, and the procol was approved by the Ethics Committee of AOU Citta’ della Salute e della Scienza, Turin Italy (Project identification code: 0089578). The trial was registered in the internal repository for clinical trials at AOU Citta’ della Salute e della Scienza, Turin, Italy (Project identification code: 1190/2016).

### 2.2. Study Design and Sample Size Determination

A one-armed optimal two-stage Simon’s design was selected, to test the hypothesis that treatment modality under investigation (BM-sparing IMRT) would increase by 20% the rate of G0–G2 (vs G3–G4) acute HT over the historical data obtained with the IMRT treatment as delivered within the RTOG 0529 trial, where the observed rate of ≥G3 acute HT was as high as 58% (rate for G0–G2: 42%) and no targeted optimization toward BM was employed, [null hypothesis (H_0_): no difference in acute HT between treatment modalities] [13]. The present study was based on the following assumptions: 1—the historical data of success (p0) was represented by the 42% rate of G0–G2 acute skin toxicity (G3–G4: 58%) detected in the RTOG 0529 study; 2—the threshold of successful trial (p1) with the treatment schedule under investigation (BM-sparing IMRT) was set to 62% of G0–G2 acute HT (G3–G4: 38%); 3—the α error (one-sided type I error) was set at 5%; 4—the β error at 20% (type II error; power 80%). At the first stage, among 21 enrolled patients, at least 9 (43%) should have been scored as G0–G2 acute HT to further proceed with the trial. At the second stage, another 18 patients will be accrued for an overall sample size of 39 patients. A minimum of 21/39 (54%) with G0–G2 toxicity represents the threshold for the final rejection of H_0_ and the fulfilment of the criteria for the definition of a “promising” treatment for the BM-sparing IMRT approach.

### 2.3. Radiotherapy Protocol

Patients underwent virtual simulation in supine position with indexed shaped knee rest and ankle support (CIVCO Medical Solutions, Kalona, IA, USA). A 3 mm slice thickness planning computed tomography was performed from the top of L1 vertebral body to the mid-femur and an isocenter found on virtual simulation. The gross tumor volume (GTV) consisted of all macroscopic foci of disease (both primary tumor and nodes) as outlined on computed tomography, accounting for information derived from ^18^FDG-PET and magnetic resonance after deformable registration. These volumes were then isotropically expanded, adding 20 mm and 10 mm respectively, to generate the corresponding clinical target volumes (CTVs), after editing to exclude surrounding osseous and muscular tissues. The elective CTV encompassed the mesorectum and appropriate draining lymphatic regions including inguinal, external and internal iliac, obturator, presacral, and perirectal/perianal nodes. Lymphatic areas were contoured as an 8–10 mm isotropic expansion around regional vessels and edited to exclude bones and muscles. A subsequent 10 mm isotropic margin was added to generate the consequential planning target volume (PTV) [14,15]. Radiotherapy strategy and dose prescription were set following the RTOG 0529 indications based on clinical stage at presentation. Patients with cT2N0 disease were given 50.4 Gy/28 fractions (1.8 Gy daily) to the primary tumor, while elective nodes were prescribed 42 Gy/28 fractions (1.5 Gy/daily). Patients presenting with cT3–T4/N0–N3 disease were prescribed 54 Gy/30 fractions (1.8 Gy daily) to the GTV, while gross nodal disease was prescribed 50.4 Gy/30 fractions (1.68 Gy daily) if sized ≤3 cm or 54 Gy/30 fractions (1.8 Gy/daily) if >3 cm. Elective nodal volume was prescribed 45 Gy/30 fractions (1.5 Gy/daily) [4,16].

### 2.4. Bone Marrow Delineation on Planning CT

The external contour of pelvic bone marrow (PBM) was outlined on the planning computed tomography employing bone windows as first described by Mell et al. [17]. The PBM was delineated as a whole and then divided into 3 subsites: (a) the iliac BM (IBM), extending from the iliac crests to the upper border of femoral head; (b) lower pelvis BM (LPBM), accounting for bilateral pube, ischia, acetabula and proximal femura, from the upper limit of the femoral heads to the lower limit of the ischial tuberosities and (c) lumbosacral BM (LSBM), extending from the superior border of L5 somatic body.

### 2.5. Active Bone Marrow Delineation on ^18^FDG-PET

**Figure 1:**
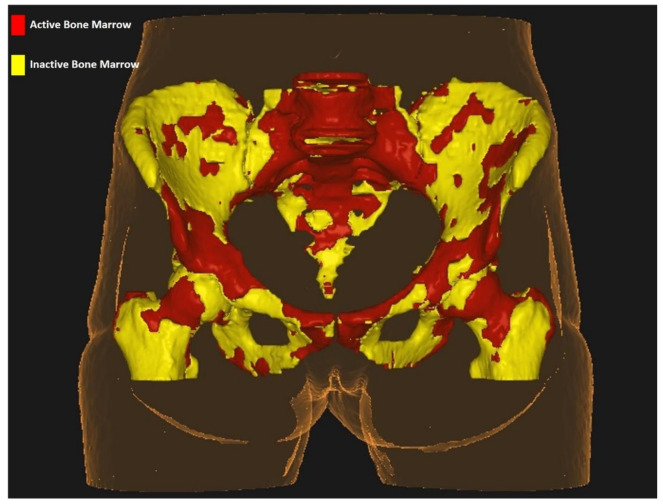
Active bone marrow segmentation within the pelvis.

All images derived from planning computed tomography were exported on the Velocity platform (Varian Medical Systems, Palo Alto, CA, USA) together with treatment volumes, organs at risk, and dose references. Given that ^18^FDG-PET images were acquired separately, we performed a rigid co-registration between computed tomography and ^18^FDG-PET. Moreover, ^18^FDG-PET standardized uptake values (SUVs) were calculated for PBM volumes, after correcting for body weight. To standardize SUVs among all patients, we normalized BM and liver SUVs. We defined as active bone marrow (^ACT^BM), the volume with higher SUV values than the SUV_mean_ for each patient, rather than the whole cohort, as proposed by Rose et al. [11]. The areas identified with the method described above were outlined within PBM as a whole and named ^ACT^BM and within each of the 3 subregions identified on planning computed tomography (LSBM, IBM, LPBM) and named ^ACT^LSBM, ^ACT^IBM, ^ACT^LPBM, respectively. Inactive BM (^INACT^BM) was identified as the difference between BM volumes as defined on planning computed tomography (PBM) and ^ACT^BM. The same procedure was repeated for all 3 subregions to identify inactive BM within all of them. The 3 volumes were hence called ^INACT^LSBM, ^INACT^IBM, ^INACT^LPBM. Figure 1 highlights ^ACT^BM (red) and ^INACT^BM as identified with the use of ^18^FDG-PET in a specific patient included in the study.

### 2.6. Planning Process and Delivery

All treatment plans were generated using the Elekta Monaco treatment planning system (version 5.51), allowing for optimization with biological cost-functions for both PTV and organs at risk. A volumetric modulated arc therapy (VMAT) approach was used, based on a single-arc of 360° (starting from180°) or, more recently, on a dual-arc technique. VMAT is ad advanced form of IMRT which combines a rotational geometry and beam modulation achieved by continuous modulation of multileaf collimator, dose rate variations and gantry rotational speed dynamics [5].

**Figure 2:**
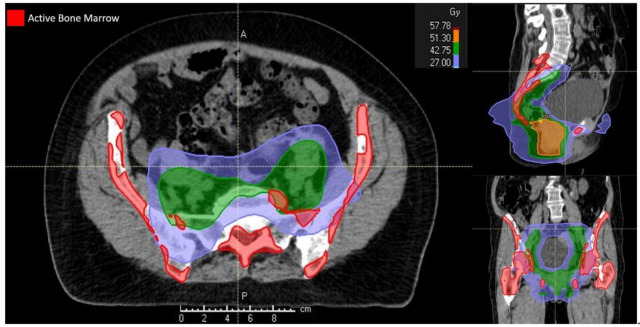
Isodose distribution for a bone marrow-sparing intensity-modulated radiotherapy treatment plan.

The planning strategy used, typically based on a pre-defined wishlist of clinical objectives and priorities, was integrated by the use of global parameters such as priorities between targets and organs at risk, dose fall-off, maximum dose and cold spot management. The standard organs at risk considered in the optimization process were bladder, external genitalia, large and small bowel and femoral heads in accordance with Kachnich et al. [4,16]. Dose constraints for active pelvic BM were targeted to both PBM and LSBM, as previously reported [9,18,19]. Table 1 reported the full set of dose constraints employed during the planning process. The treatment was finally delivered using the Elekta Synergy platform. Figure 2 shows the isodose distribution for a BM-sparing IMRT plan delivered to a patient in the present study.

### 2.7. Chemotherapy

Patients received concomitant CHT, consisting of 5-fluorouracil (1000 mg/m^2^/day) given as continuous infusion for 96 h (days 1–5 and 29–33) combined with mitomycin C (10 mg/m^2^) given as bolus (days 1 and 29). A total of 2 concurrent cycles were planned for each patient. CHT discontinuation or drugs modification were planned in case of major toxicities.

### 2.8. Toxicity Evaluation and Clinical Assessment

Acute gastro-intestinal (GI), genitourinary (GU), dermatologic, hematologic, and genital toxicities were assessed during treatment and scored according to the Common Toxicity Criteria for Adverse Events scale v4.0 (CTCAEv4.0) [20]. The worst grade toxicity for each category observed within 90 days from treatment end was recorded as an acute toxicity event. All toxicities occurring >90 days from RT discontinuation were classified as late toxicity. During follow-up, patients had a clinical examination with digital rectal exam and anoscopy evaluation at 4, 8, and 12 weeks. At 12 weeks, pelvic MRI and ^18^FDG-PET were performed and a biopsy take in case of suspicious persistent disease. Re-evaluation at 26 weeks from the start of treatment was done with anoscopy, eventual biopsy, and ^18^FDG-PET. A complete response was defined in case of negativity of the pathology examination at biopsy. A salvage abdomino-perineal resection was recommended for persistent disease (at pathology) or for locally progressive or recurrent disease (at imaging and pathology).

### 2.9. Hematologic Toxicity Evaluation

All patients underwent a weekly complete blood count. HT was graded according to CTCAEv4.0 grading system [20]. Endpoints evaluated in the present analysis were white blood cell count (WBC), absolute neutrophil count (ANC), hemoglobin (Hb) and platelet (Plt) count nadirs after each CHT cycle and the highest-grade toxicity for all blood cells. HT was defined as each hematologic event with a grade ≥3.

**Figure 3:**
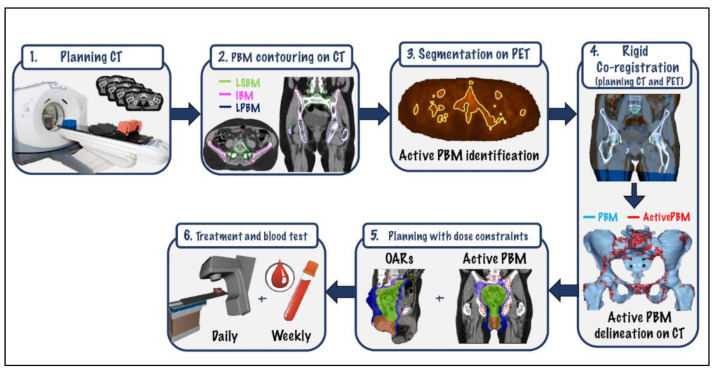
Comprehensive workflow for BM-sparing IMRT. CT: computed tomography; PBM: pelvic bone marrow; LSBM: lumbar-sacral bone marrow; IBM; iliac bone marrow; LPBM: lower-pelvic bone marrow; PET: positron-emission tomography; OARs: organs at risk.

Figure 3 shows a complete outline of the different steps taken to deliver BM-sparing IMRT to the enrolled patients and to test acute HT

## 3. Results

A total of 21 patients were included in the first step of this prospective phase II trial. Detailed patient characteristics are shown in Table 2. Mean age was 64 (range 29–81) and patients were mainly female (71%), HIV-negative (90%), with an anal canal primary tumor (90%), T2–T3 stage (76%), N1 nodal disease (70%) and global stage IIIC (43%). None of them underwent a preventive colostomy (100%). Patients were mainly treated with a dual-arc VMAT approach (86%), up to a total dose to the primary tumor PTV of 54 Gy (95%) and to 45 Gy (95%) to the prophylactic volumes delivered in conventional fractionation. A total of 12 node positive patients, also received a simultaneous integrated boost to the macroscopic nodal disease up to 50.4 Gy. Mean radiotherapy duration time was 46 days. Patients undergoing a treatment break ≥3 days were 9%. All but 1 patient were submitted to 2 cycles of CHT with no dose reduction during treatment. See Table 3 for details.

### Acute Hematologic Toxicity and Dosimetric Outcomes

**Figure A1:**
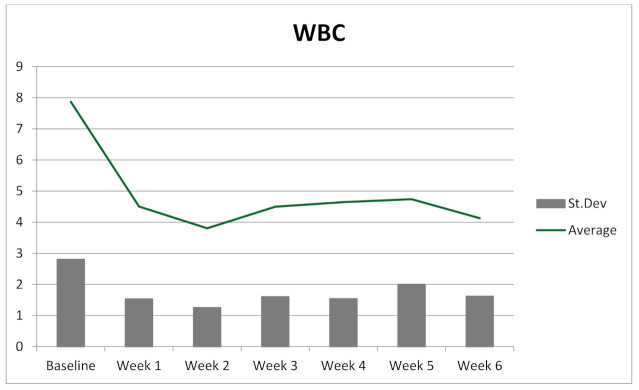
White blood cell count trend during treatment.

**Figure A2:**
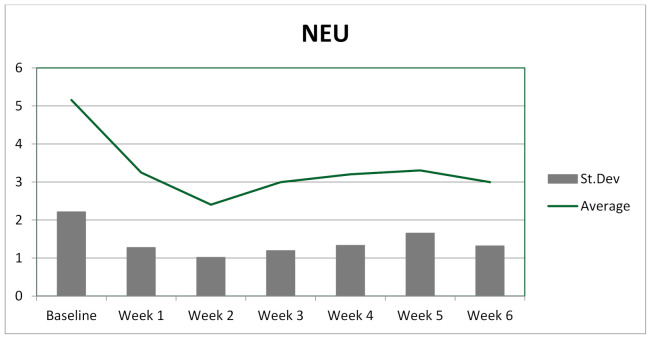
Nutrophil count trend during treatment.

**Figure A3:**
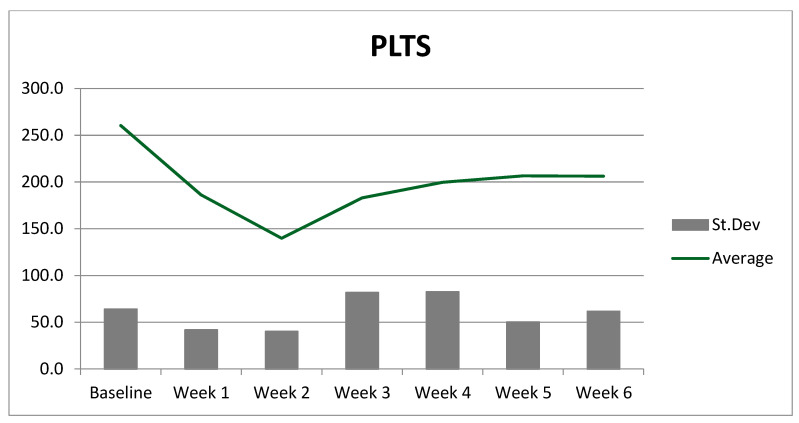
Platelet count trend during treatment.

**Figure A4:**
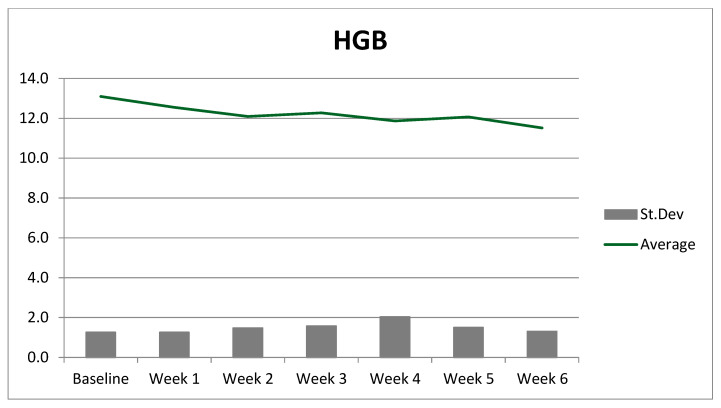
Hemoglobin level trend during treatment.

Mean value at baseline for WBC was 7.860/cm^3^ [Standard Deviation (SD) SD:2.800], which dropped to a minimum of 3.800/cm^3^ (SD:1.260) at Week 2, reaching 4.130/cm^3^ (SD:1.620) at the end of treatment (Week 6). ANC at baseline was 5.160/cm^3^ (SD:2.210), which dropped to a minimum of 2.400/cm^3^ (SD:1.010) at Week 2, reaching 2.990/cm^3^ (SD:1.314) at Week 6. Mean value at baseline for Plts was 260.000/cm^3^ (SD:64.000), which dropped to a minimum of 140.000/cm^3^ (SD:40.000) at Week 2, reaching 206.000/cm^3^ (SD:61.000) at Week 6. For Hb, mean value at baseline was 13.1 g/dL (SD: 1.3), which dropped to a minimum of 11.5 g/dL (SD:1.3) at Week 6. Appendix A shows the weekly trend for the analyzed blood parameters during concurrent RT-CHT (Figure A1, Figure A2, Figure A3 and Figure A4).

Major non-hematologic toxicities comprised G3 events for skin and genitalia in 14% and 5% of patients, respectively (Table 4).

Maximum detected acute HT comprised 9% of patients experiencing leukopenia ≥ G3 and 5% with neutropenia ≥G3. Grade 3 anemia was observed in 5% of patients, while no G3 thrombocytopenia was detected. Up to 24% experienced G2 anemia and leukopenia and 9% G2 thrombocytopenia (Table 4). Overall, only 4 out of 21 patients treated (19%) experienced ≥G3 acute HT. That means that 17 patients (81%) experienced G0-G2 events, with a threshold set by the trial design at 9 patients (43%) at least.

Dosimetric parameters pertinent to both treatment volumes and standard organs at risk are shown in Table 5. Those relative to active BM are presented in Table 6. Appendix B shows dosimetric parameters for both pelvic bones and inactive BM (Table A1 and Table A2).

## 4. Discussion

Concurrent CHT-RT is the standard of care in patients affected with anal cancer, improving clinical outcomes over exclusive radiation alone as shown in the ACT-I and EORTC22861 trials [21,22]. Intensified CHT regimens are more effective compared to mono-chemotherapy regimens as observed in the intergroup study [23]. In first generation trials, RT was delivered employing 2- or 3-dimensional solutions, with the consequent effect that a consistent amount of normal tissues was exposed to unintended irradiation, with organs at risk such as bladder, bowel, perineal region and BM included within treatment fields to receive medium to high RT doses [3,21]. This led to a non-negligible toxicity profile as in the standard arm of the RTOG 9811 trial, where ≥G3 gastro-intestinal events were observed in 35% of patients, while ≥G3 acute HT was seen in up to 61% [3].

In particular, HT may have detrimental clinical repercussion on anal cancer patients, hampering their overall compliance to treatment and jeopardizing the oncological outcomes [9]. Even in more recent series, employing full course IMRT, acute HT was relevant with rates of ≥G3 events as high as 58%, as in Salama et al. and in the RTOG 0529 trial, particularly in case of lack of adoption of selective avoidance of the hematopoietic regions [4,24]. Bone marrow is a crucial dose-limiting cell renewal tissue for wide-field irradiation [6]. Since BM stem cells are exquisitely radiosensitive, RT has an important myelosuppressive effect, causing apoptosis and stromal damage, with peculiar pathologic and radiographic modification [6]. The major functional sites for BM in the adult population are the pelvis and lumbar vertebrae which account for approximately 60% of the body amount. Pelvic bones may contain up to 40% of the total functional BM [6,11]. This is the reason pelvic irradiation can be a contributing factor in determining HT in anal cancer patients during combined modality treatment. The extent of radiation-induced bone marrow damage has been demonstrated to be correlated with both radiation dose and BM volume receiving irradiation [7,8,9,10,11]. In this sense, we decided to test, within a prospective phase II trial, the hypothesis that selectively sparing BM comprised within pelvic bones may decrease the acute HT profile among anal cancer patients undergoing concurrent RT-CHT for squamous cell carcinoma of the anus.

The first procedural step to implement BM-sparing IMRT is the delineation of the bone marrow. Several approaches have been employed such as the use of the external surface of pelvic bones as a surrogate for BM, as in the RTOG 0418 trial [25]. Other authors outlined the marrow cavity, corresponding to the lower Hounsfield Unit part of an osseous segment as seen on computed tomography imaging [8]. Since functional imaging may be a useful tool in defining active BM within an osseous segment, potentially providing a more accurate spatial definition and a patient-specific localization, we employed ^18^FDG-PET to identify ^ACT^BM (red marrow—involved in the hematopoietic process) and to differentiate it from the inactive marrow (yellow marrow—made of fat tissue) [6]. This is relevant, since BM distribution within bones can vary, depending on gender and age. As an example, Campbell et al. investigated BM distribution according to ^18^F-FLT-PET in a cohort of 51 lung cancer patients. Women had a higher proportion of functional BM in the pelvis, proximal femurs and skull, while men in the sternum and ribs, clavicles and scapulae. Elderly patients (>75 years) had a higher relative proportion of active BM in the ribs, clavicles, and scapulae [26]. The use of ^18^FDG-PET may be advantageous since it provides a reliable picture of individual BM distribution, allowing for accurate definition and localization, with a potential volume reduction compared to the use of the whole bone as a surrogate for BM. This may decrease the challenges experienced during treatment planning due to the simultaneous need for target coverage and avoidance of non-hematopoietic organs at risk such as bladder and bowel, facilitating the trade-off with the dose constraints targeting BM.

As in Rose et al., we employed the SUV_mean_ calculated within BM for each patient to define and contour the ^ACT^BM subregions [10]. The use of a patient-specific BM SUV threshold instead of a population-based modality represents a control tool towards eventual differences in terms of imaging process across different platforms and in terms of BM SUV values according to gender and age categories [10].

The second step of BM-sparing IMRT implies the inclusion of ^ACT^BM as an organ at risk in the optimization process during treatment planning [27]. The combination of dose constraints was addressed to both low dose to PBM and medium-high doses to LSBM. The influence of low doses to PBM in determining a decrease in blood cell nadirs and the occurrence of acute HT has been documented in anal and cervical cancer patients where subjects reporting PBM V_10_ ≥ 90 and PBM V_20_ ≥ 75% were shown to have worse HT [7,17]. Hence, PBM V_10_ and V_20_ ≤ 90% and 75%, respectively, were used in our study, as in the INTERTECC-2 trial, which explored BM-sparing IMRT in cervical cancer patients [18]. With respect to LSBM, it has been shown that the relative proportion of ^ACT^BM within LSBM is as high as 67% [11]. Moreover, ^ACT^LSBM is centrally located and usually in close proximity to treatment volumes pertinent to both primary tumor and macroscopic nodes, with a higher likelihood to receive medium-high dose radiation if not properly taken into account [28]. That makes this structure relevant for the occurrence of HT during combined CHT-RT in anal cancer. We previously demonstrated that LSBM-V_40_ was correlated with a higher likelihood to develop ≥G3 HT in anal cancer patients [9,11]. Moreover, according to Lyman-Kutcher-Burman modeling, we outlined that LSBM mean dose should be kept <32 Gy to minimize >G3 HT rates in a similar population [19]. Hence, we also set ^ACT^LSBM-V_40_ < 41 % and ^ACT^LSBM-D_mean_ < 32 Gy as constraints in our treatment planning process to reduce HT. This approach also allowed to minimize the interplay effect between low dose to PBM and the tolerance threshold of LSBM to RT, as demonstrated by our group [19].

The performance of BM-sparing IMRT in terms of dosimetric outcomes was found to be robust, with respect to both target coverage and normal tissue avoidance. Standard organs at risk, such as bladder, bowel, external genitalia and femoral heads were adequately spared as average dose objectives were consistently met. With respect to hematopoietic structures, the dosimetric requirements were met on average for both ^ACT^PBM (V_10_ and V_20_) and ^ACT^LSBM (V_40_ and mean dose).

The acute toxicity profile was generally mild, with non-hematologic major toxicities (≥G3) being rather contained (skin: 14%; genitalia: 5%) and, interestingly no major gastro-intestinal events recorded. With respect to hematologic toxicity, only 4 out of 21 patients treated (19%) experienced ≥ G3 acute HT. This data is rather promising, since in our previous studies, reporting on patients treated with VMAT and image-guided IMRT delivered with no selective avoidance of BM, ≥G3 acute HT was consistently above 25% [5,29].

In the first step of this prospective phase II study, up to 17 patients (81%) experienced G0-G2 acute HT, way above the threshold set at 9 patients (43%). As per the trial design, these results prompted us to continue with the second step of this prospective phase II trial to reject the null hypothesis (no difference in acute HT between standard and BM-sparing IMRT) and to potentially fulfil the criteria to define BM-sparing IMRT as a “promising” treatment for anal cancer patients undergoing concurrent CHT-RT with definitive intent to reduce the acute hematologic toxicity profile.

## 5. Conclusions

The first step of this prospective phase II trial highlighted the feasibility of all the phases of the BM-sparing IMRT approach, including ^ACT^BM segmentation based on ^18^FDG-PET, co-registration with simulation computed tomography and targeted optimization during the treatment planning process. The whole package was robustly implemented in clinical practice. Clinical results were found to be promising, with a detectable reduction in the acute HT profile, compared to historical data, >prompting us to conclude the second part of this prospective phase II trial.

## Figures and Tables

**Table 1:** Dose constraints employed in the optimization process during treatment planning.

Structure	Parameter	Goal
**PTV**	D_95%_	≥95%
	D_max_	≤107%
**Bladder**	V_30_	<50%
	V_40_	<35%
	V_50_	<5%
**Large Bowel**	V_30_	<200 cm^3^
	V_35_	<150 cm^3^
	V_40_	<20 cm^3^
	D_max_	<50 Gy
**Small Bowel**	V_30_	<200 cm^3^
	V_35_	<150 cm^3^
	V_40_	<20 cm^3^
	D_max_	<50 Gy
**External Genitalia**	V_20_	<50%
	V_30_	<35%
	V_40_	<5%
**Femoral Heads**	V_30_	<50%
	V_40_	<35%
	V_50_	<5%
**Active PBM**	V_10_	<90%
	V_20_	<75%
**Active LSBM**	V_40_	<41%
	D_mean_	<32 Gy

Legend: PTV: planning target volume; PBM: pelvic bone marrow; LSBM: lumbar-sacral bone marrow.

**Table 2:** Patient and tumor characteristics.

Variable	N (%)
**Age**	
Mean	64
Range	29–81
**Sex**	
Female	15 (71)
Male	6 (29)
**HIV status**	
Positive	2 (10)
Negative	19 (90)
**Primary tumor site**	
Anal canal	19 (90)
Anal margin	1 (5)
Both	1 (5)
**T-stage**	
T1	1 (5)
T2	8 (38)
T3	8 (38)
T4	3 (14)
**N-stage**	
N0	6 (29)
N1	15 (71)
**Global stage**	
I	0 (0)
IIA	5 (24)
IIB	1 (5)
IIIA	6 (28)
IIIB	0 (0)
IIIC	9 (43)
**Grading**	
G1	0 (0)
G2	4 (19)
G3	3 (14)
NA	14 (67)

Legend: N: number; T-stage: tumor stage; N-stage: nodal stage; NA: not available.

**Table 3:** Treatment characteristics.

Variable	N (%)
**VMAT approach**	
Single-arc	3 (14)
Dual-arc	18 (86)
**PTV dose-tumor (Gy)**	
54 Gy/30 fractions	20 (95)
50.4 Gy/28 fractions	1 (5)
**PTV dose-positive nodes (Gy)- 12 pts**	
54 Gy/30 fractions	0 (0)
50.4 Gy/30 fractions	12 (100)
**PTV dose-negative nodes (Gy)**	
45 Gy/30 fractions	20 (95)
42 Gy/30 fractions	1 (5)
**Chemotherapy**	
5-FU + MMC	21 (100)
**Cycles**	
1	1 (5)
2	20 (95)
**Chemotherapy dose reduction**	
Yes	1 (5)
No	20 (95)
**RT duration (days)**	
Mean	46
Range	38–77

Legend: IMRT: intensity-modulated radiotherapy; PTV: planning target volume; N: number; 5-FU: 5-fluorouracil; MMC: mytomicin C; RT: radiotherapy.

**Table 4:** Acute toxicity profile.

Acute Toxicity	G0	G1	G2	G3	G4
Skin	0 (0)	38 (0)	10 (48)	3 (14)	0 (0)
Gastro-intestinal	7 (33)	6 (29)	8 (38)	0 (0)	0 (0)
Urinary	6 (28)	10 (48)	5 (24)	0 (0)	0 (0)
Genitalia	9 (43)	7 (33)	4 (19)	1 (5)	0 (0)
Anemia	13 (62)	5 (24)	2 (9)	1 (5)	0 (0)
Leukopenia	5 (24)	5 (24)	8 (38)	2 (9)	1 (5)
Neutropenia	11 (53)	2 (9)	5 (24)	1 (5)	2 (9)
Thrombocytopenia	17 (82)	2 (9)	2 (9)	0 (0)	0 (0)

**Table 5:** Dosimetric parameters for both target volumes and organs at risk.

**PTV**				
**Structure**	**Parameter**	Mean	SD	
	D_98_ (Gy)-50.4 Gy	48	0	
PTV-tumor	D_2_ (Gy)-50.4 Gy	54	0	
	D_98_ (Gy)-54 Gy	50.2	2.44	
	D_2_ (Gy)-54 Gy	57.55	1.36	
	V_95_ (%)	95.45	8.61	
	V_107_ (%)	2.66	2.81	
PTV-elective volumes	D_98_ (Gy)-42 Gy	48	0	
	D_2_ (Gy)-42 Gy	54	0	
	D_98_ (Gy)-45 Gy	50.2	2.44	
	D_2_ (Gy)-45 Gy	57.55	1.36	
	V_95_ (%)	95.45	8.61	
	V_107_ (%)	2.66	2.81	
**OARs**				
**Organ at risk**	**Parameter**	Mean	SD	
Bladder	V_30_ (%)	42.95	14.3	
	V_40_ (%)	18.67	15.76	
	V_50_ (%)	4.33	12.31	
	D_2_ (Gy)	45.90	4.97	
	Mean dose (Gy)	29.29	4.23	
Bowel	V_30_ (cc)	88.86	108.05	
	V_35_ (cc)	63.38	78.42	
	V_40_ (cc)	19	24.65	
	V_45_ (cc)	5.90	6.99	
	D_2_ (Gy)	44.47	3.35	
	Mean dose (Gy)	18.20	5.07	
External genitalia	V_20_ (%)	39.67	24.87	
	V_30_ (%)	27.80	21.86	
	V_40_ (%)	18.14	13.57	
	D_2_ (Gy)	49.61	10.60	
	Mean dose (Gy)	21.43	10.16	
Femoral heads	V_30_ (%)	13	8.14	
	V_40_ (%)	4	5.70	
	V_45_ (%)	1	4.56	
	V_50_ (%)	1	4.35	
	D_2_ (Gy)	40	5.10	
	Mean dose (Gy)	17	3.42	

Legend: PTV: planning target volume; OAR: organs at risk; cc: cubic centimeters; SD standard deviation; Gy: Gray.

**Table 6:** Dosimetric parameters for pelvic active bone marrow.

Structure	Parameter	Results		Structure	Parameter	Results	
		Mean	SD			Mean	SD
**^ACT^PBM**	D_mean_ (Gy)	25.57	3.17	**^ACT^IBM**	D_mean_ (Gy)	21.76	3.74
	V_5_	95.48	5.94		V_5_	94.00	8.26
	V_10_	86.09	8.84		V_10_	80.43	12.05
	V_15_	75.52	9.59		V_15_	66.95	13.81
	V_20_	64.24	9.16		V_20_	52.62	14.03
	V_30_	40.14	8.90		V_30_	25.61	11.75
	V_40_	17.62	6.58		V_40_	8.23	6.38
	V_45_	4.38	4.05		V_45_	1.33	2.61
	V_50_	0.57	1.28		V_50_	0.14	0.48
**^ACT^LSBM**	D_mean_ (Gy)	29.90	4.98	**^ACT^LPBM**	D_mean_ (Gy)	27	3.14
	V_5_	93.67	9.43		V_5_	100	0.00
	V_10_	88.76	12.87		V_10_	91.57	8.13
	V_15_	83.52	14.43		V_15_	79.61	12.60
	V_20_	76.19	14.78		V_20_	67.38	12.13
	V_30_	55.86	13.36		V_30_	41.24	10.00
	V_40_	28.57	10.84		V_40_	18.28	7.11
	V_45_	6.90	6.04		V_45_	5.67	5.01
	V_50_	1.00	2.28		V_50_	0.71	1.70

Legend: Gy: Gray; SD: standard deviation; ^ACT^PBM: active pelvic bone marrow; ^ACT^IBM: active iliac bone marrow; ^ACT^LSBM: active lumbar-sacral bone marrow; ^ACT^LPBM: active lower pelvic bone marrow.

## Appendix B

**Table A1:** Dosimetric parameters for pelvic bones and their subsites as outlined using the outer contours of the osseous segments.

Structure				Structure			
	**Parameter**	Mean	SD		**Parameter**	Mean	SD
**PBM**	D_mean_	24.76	2.47	**IBM**	D_mean_	33.19	56.66
	V_5_	95.80	4.67		V_5_	92.71	12.82
	V_10_	85.48	7.53		V_10_	80.23	11.80
	V_15_	72.33	8.24		V_15_	66.95	12.82
	V_20_	59.67	7.90		V_20_	52.57	14.37
	V_30_	36.19	7.27		V_30_	27.38	17.51
	V_40_	16	5.08		V_40_	12.09	19.21
	V_45_	4	3.20		V_45_	5.66	19.88
	V_50_	0.38	0.97		V_50_	4.43	20.07
**LSBM**	D_mean_	30.15	4.30	**LPBM**	D_mean_	24.28	2.74
	V_5_	94.24	7.64		V_5_	99.19	2.87
	V_10_	89.80	10.48		V_10_	87.71	9.90
	V_15_	84.23	11.77		V_15_	70.76	10.45
	V_20_	76.86	12.65		V_20_	56.90	9.34
	V_30_	56.47	11.85		V_30_	34.04	8.54
	V_40_	29.52	9.90		V_40_	14.43	4.92
	V_45_	7.80	6.78		V_45_	3.95	2.76
	V_50_	1.05	2.54		V_50_	0.33	0.79

**Table A2:** Dosimetric parameters of inactive bone marrow as outlined on positron emission tomography.

Structure				Structure			
	**Parameter**	Mean	SD		**Parameter**	Mean	SD
**1-PBM PET**	D_mean_	24	2.90	**1-IBM PET**	D_mean_	21	3.44
	V_5_	96	3.96		V_5_	91	11.11
	V_10_	85	7.26		V_10_	79	11.75
	V_15_	70	8.87		V_15_	65	12.65
	V_20_	56	10.15		V_20_	48	13.32
	V_30_	33	9.68		V_30_	22	8.99
	V_40_	14	5.67		V_40_	8	5.23
	V_45_	4	2.92		V_45_	1	1.64
	V_50_	0.2	0.68		V_50_	0	0
**1-LSBM PET**	D_mean_	31	4.70	**1-LPBM PET**	D_mean_		
	V_5_	96	5.39		V_5_	99	3.32
	V_10_	93	8.88		V_10_	87	10.61
	V_15_	85	11.99		V_15_	68	11.41
	V_20_	76	15.50		V_20_	53	11.42
	V_30_	56	15.66		V_30_	30	9.76
	V_40_	31	10.42		V_40_	11	4.87
	V_45_	11	9.56		V_45_	3	1.80
	V_50_	2	3.77		V_50_	0.1	3.06
